# In Vivo Toxicity of Silver Nanoparticles and Silver Ions in Zebrafish (*Danio rerio*)

**DOI:** 10.1155/2012/293784

**Published:** 2011-12-01

**Authors:** Katrine Bilberg, Mads Bruun Hovgaard, Flemming Besenbacher, Erik Baatrup

**Affiliations:** ^1^Interdisciplinary Nanoscience Center (iNANO) and Department of Physics and Astronomy, Aarhus University, 8000 Aarhus C, Denmark; ^2^Section of Zoophysiology, Department of Biological Sciences, Aarhus University, C.F. Moellers Allé 3, Building 1131, 8000 Aarhus C, Denmark

## Abstract

The influence of water chemistry on characterised polyvinyl pyrrolidone- (PVP-) coated silver nanoparticles (81 nm) was investigated. NaCl solution series of 100–800 mg L^−1^ lead to initial and temporal increase in nanoparticles size, but agglomeration was limited. pH variation (5–8) had only minor influence on the hydrodynamic particle size. Acute toxicity of nanosivler to zebrafish (*Danio rerio*) was investigated in a 48-hour static renewal study and compared with the toxicity of silver ions (AgNO_3_). The nanosilver and silver ion 48-hour median lethal concentration (LC_50_) values were 84 **μ**g L^−1^ and 25 **μ**g L^−1^, respectively. To investigate exposure-related stress, the fish behaviour was observed visually after 0, 3, 6, 12, 24, 27, 30, and 48 hours of both nanosilver and ionic silver treatments. These observations revealed increased rate of operculum movement and surface respiration after nanosilver exposure, suggesting respiratory toxicity. The present study demonstrates that silver nanoparticles are lethal to zebrafish.

## 1. Introduction

Silver is a rarely occurring element in the earth's crust (0.05–0.1 ppm) but is deposited at much higher concentrations in ores in association with other elements [1]. In the aquatic environment, silver originates from leaching, mining, or anthropogenic sources [2]. It is traditionally incorporated in, for example, coins, jewellery, electronics, and photographic manufacturing [3]. In addition, the antibacterial capacity of both ionic silver and nanosilver has expanded its use significantly [3, 4], being incorporated in a variety of products, including clothing, paints, plastics, food containers packaging, wound dressings, bandages, and household appliances such as refrigerators and washing machines [3, 5].

The concentration of silver has been found to be less than 5 ng L^−1^in three Connecticut undeveloped headwaters and between 25 and 100 ng L^−1^ in rivers from industrialised and urban areas [6]. In rivers in Texas, particulate silver concentrations range from <0.01 to 62 ng L^−1^[7]. Generally, the concentration of silver ions is extremely low in surface waters, because ionic silver binds to a variety of negatively charged ligands [2, 8]. The concentration of silver nanoparticles from consumer products in the aquatic environment is predicted to be about 0.01 *μ*g L^−1^[9]. In 2010, the silver concentration in courses of the Rhine, receiving outlets of textiles and plastics containing nanosilver, was estimated to be between 4 and 40 ng silver L^−1^, accounting for 15% of the total silver release [10]. Future discharge of nanosilver to the aquatic environment will undoubtedly increase seriously due to the expected extensive use of nanosilver.

The silver ion is toxic to fish [11–13], and therefore it is crucial also to establish the toxicity of nanosilver. Metal nanoparticles of cupper have been demonstrated to be acutely toxic to zebrafish with an LC_50_ value of 1–1.5 mg L^−1^ [14, 15]. In contrast, aluminium, cobalt, nickel, and titanium dioxide nanoparticles were found to be less toxic to adult zebrafish with a 48-hour median lethal concentration (LC_50_) >10 mg L^−1^[14].

Metal nanoparticles possess unique properties due to their size, shape, surface structure, aggregation characteristics, and chemical composition [16–18] that differ from their respective soluble metal. How water chemistry such as salinity and pH influence the toxicity of nanoparticle is sparsely investigated [19] but possibly affects the size and shape of the particles [20]. Consequently, it is important to characterize the nanoparticles in the actual environment in order to know how they chemically interact as this likely alters their toxicity.

The aim of this study was to establish the 48-hour lethal concentration (LC_50_) of waterborne 81 nm mean size silver nanoparticles to zebrafish (*Danio rerio*) and to compare the toxicity of these silver nanoparticles with silver ions (administered as silver nitrate). In addition, the effects of water chemistry on nanosilver characteristics were investigated.

## 2. Methods

### 2.1. Experimental Animal

Zebrafish (*Danio rerio*) were purchased from Credo Fish (Aalborg, Denmark). The fish were acclimatised for at least two weeks in a 26°C stock aquarium containing aerated head tank water. Head tank water consists of demineralised water mixed with nonchlorinated tap water- (16 : 1) added NaCl to a conductivity of approximately 275 *μ*S (132 mg L^−1^), which is optimal for zebrafish according to Nüsselin-Volhard and Dahm [21], and also prevents fungi growth. The concentrations (in mg L^−1^) of the predominant ions in the head tank water were: SO_4_^2−^ 4.25, Na^+^ 43.19, NO_3_^−^ 0.09, Mg^2+^ 0.75, K^+^ 0.21, Cl^−^ 63.93, Ca^2+^ 5.31, and HCO_3_^−^ 19.31, in total ~137 mg L^−1^. The photoperiod was 14 : 10 (light : dark) where the light period was initiated by an artificial sunrise. The sunrise was made by gradually increasing the voltage to a weak luminous lamp ten minutes before the luminous intensity rose in the room. The aquaria were illuminated with 95–105 LUX from an artificial light source.

### 2.2. Preparation of Silver Nanoparticle Suspension and Ionic Silver Solution

Silver nanoparticle powder (stated by the manufacturer to be spherical 30–40 nm particles), coated with 0.2% polyvinyl pyrrolidone (PVP) and a purity of 99.5%, was purchased from NanoAmor (Houston, USA). PVP-coated silver nanoparticles were chosen, as they are easily dispersed in water. A water dispersion of silver nanoparticles was prepared by suspending 0.5 g silver nanoparticle powder in 100 mL Milli-Q water, followed by immediate ultra sonication (Ultrasonic Homogenizer, BioLogics, Inc, Virgina, USA) in order to deaggregate the suspension. The sonicator was mounted with a solid titanium tip with a diameter of 9.5 mm. The suspension was placed on a magnetic stirrer and sonicated four times of 15 min over a 2-hour time span with pulses of half a second duration. The power output was 100 Watt and the output frequency was 20 kHz. Following sonication, the suspension was centrifuged at 1000 g for 2 hours (Sigma 3k 30, Struers KEBO lab., Demark), and finally the supernatant was filtrated through a 0.2 *μ*m mixed cellulose ester filter (Frisenette ApS, Denmark). The stock nanosilver suspension had a clear yellow colour and was stored in darkness at 6°C until use.

To determine the concentration of silver in the stock nanosilver suspension, equal volumes of the suspension and 69% nitric acid (HNO_3_) were mixed resulting in dissolution of the nanosilver. This solution was then diluted with Milli-Q water until the silver concentration was within the linear measuring range of the Atomic Absorption Spectrophotometer (AAS). The concentrations of silver were determined using a Perkin-Elmer Aanalyst 300 AAS (Perkin-Elmer, Hvidovre, Denmark) mounted with a silver lumina hollow cathode lamp (Perkin-Elmer, Hvidovre, Denmark). In all cases, double measurements were performed, and the instrument detection limit for silver was 36 *μ*g L^−1^.

Free silver ions in the silver nanoparticle stock suspension were measured by a silver ion-selective electrode (ISE) ISE225 (Hach Lange APS, 2700 Brønshøj, Denmark). The calibration curve was constructed from diluting AgNO_3_ solutions in the silver ion range 0.1–1 mg L^−1^. The ISE is sensitive to interferences from sulphides, which, however, are not likely to be present in the suspension. The amount of dissolved silver was also determined by ultracentrifugating the stock suspension 30 min at 100 000 g (Optima L80xp centrifuge, sw55 snE17986 rotor); whereafter the silver content in the supernatant was determined by AAS. The applied methods have previously been used to determine the content of dissolved metal nanoparticles [15, 22]. Double measurements estimated that in the nanosilver suspension, approximately 40% of the silver was in the form of silver ions.

To determine if the concentration of silver changed over time in the test tanks, water samples were obtained from a tank containing fish 0, 2, and 24 hours after the application of 80 *μ*g L^−1^ silver nanoparticles. Using AAS, no decline in silver concentrations was found two hours after nanosilver application, whereas the concentration was reduced by 41% after 24 hours.

Silver nitrate (AgNO_3_) pellets (SigmaAldrich, Steinheim, Germany) with a purity >99.5% were dissolved in Milli-Q water.

### 2.3. Silver Nanoparticle Characterisation

#### 2.3.1. Powder X-Ray Diffraction

Crystallite size and crystalline phase were evaluated by powder X-ray diffraction (PXRD) using a STOE STAPI P (STOE & Cie GmbH, Darmstadt, Germany) powder diffractometer emitting CuK*α*_1_ (*λ* = 0.15405 nm ) radiation, equipped with a curved 1D-PSD detector [23]. For analysis, the silver nanoparticle powder was mixed with diluted wood glue, allowing a fixation of the highly electrostatic sample between two Mylar discs, whilst providing a mounting transparent to the incident and diffracted X-ray radiation. Diffraction profiles were inspected to determine the presence of nanocrystallite domains (i.e., if the material was amorphous or not) and to identify the crystalline phase by direct comparison to reference libraries (International Centre for Diffraction Data, PDF-2). Further, an estimate of the crystallite domain size was obtained by fitting the 111 diffraction peak to a Voigt function (Origin7.5, OriginLab Corporation, USA), using the Scherrer formula [24, 25] on the full width half maximum (FWHM) peak value, after a linear background subtraction and instrumental profile correction of both the Gaussian and Lorentzian profile components [25]. The instrumental profile broadening was estimated from the fit of a Voigt function to the diffraction line profiles of a NIST LaB6 standard sample.

#### 2.3.2. Transmission Electron Microscopy

The primary nanoparticle size and morphology were assessed using a Phillips CM20 transmission electron microscope (TEM) working at 200 keV. For TEM analysis, stock nanoparticle suspensions were diluted 1 : 100 and 10 *μ*L were pipetted onto holey carbon grids (S147-4, Plano GmbH, Germany) and subsequently left to evaporate in a laminar flow hood. A series of images was selected to manually establish size distributions by tracing single particle contours using the scanning probe image software SPIP (Image Metrology ApS, Lyngby, Denmark).

#### 2.3.3. Dynamic Light Scattering and Zeta Potential Measurements

The hydrodynamic diameter and zeta potential of the nanoparticles were characterised by dynamic light scattering (DLS) using a Malvern Zetasizer Nano (Malvern Instruments Ltd, Worcestershire, UK). Besides characterising the stock suspension (pH 3.9, Milli-Q water), a series of varying aqueous conditions were tested by mixing the stock silver nanoparticle suspension in a 1 : 1 ratio with preadjusted solutions of well-defined pH values, sodium chloride (NaCl) concentrations, or combinations thereof, 5 min prior to DLS measurements. The resulting pH and NaCl concentrations were pH 5–8 (steps of pH 1) and (100, 200, 400, 600, and 800) mg L^−1^ NaCl, respectively; with the latter NaCl series run both at pH 3.9 (stock suspension pH) and at pH 6.9 (head tank water pH). Also included in the series was head tank water. All measurements were performed at 26°C and repeated after initial (5 min), 30 min or 1 hour, 12 hours (data not shown), and 24 hours, with samples kept between measurements under conditions identical to the fish tanks used for nanosilver toxicity assessment. Each DLS measurement was run in triplicate using automated, optimal measurement time and laser attenuation settings. The recorded correlation functions and measured particle mobilities were converted into size distributions and zeta potentials, respectively, using the Malvern Dispersion Software (V5.10, http://www.zetasizer.com/).

### 2.4. Acute Toxicity Testing of Silver Nanoparticles and Silver Ions

A 48-hour acute toxicity (LC_50_) test of silver nanoparticles on adult male zebrafish (standard length 28.1 ± 0.2 mm weighing 0.42 ± 0.04 g; *n* = 110) was conducted in a static water renewal experiment, according to the Organisation for Economic Cooperation and Development (OECD) guideline for testing of chemicals (OECD, 1992). The eight silver nanoparticle nominal exposure concentrations of 18, 36, 54, 72, 89, 107, 125, and 143 *μ*g L^−1^ plus an unexposed control were determined from a preliminary exposure study. In addition, two PVP controls (Sigma-Aldrich, Steinheim, Germany) were included to assess the possible toxicity of the PVP coating. Zebrafish were exposed to PVP concentrations equalling half of the nanosilver LC_50_ value (42 *μ*g L^−1^) and hundred times the nanosilver LC_50_ value (8400 *μ*g L^−1^). Ten randomly selected male zebrafish were exposed to each concentration in 14 L aerated seamless glass tanks (29 cm × 21 cm × 23 cm) (length x width x height) containing five L of head tank water. After 24 h of treatment, the fish were transferred to new tanks containing their respective concentrations of nanosilver. The zebrafish were not fed 24 hours prior to or during the experiment in order to maintain constant exposure concentrations, given that nanoparticles might adhere to food and faeces particles [26].

A similar 48-hour acute toxicity (LC_50_) test of silver ions was conducted with zebrafish of standard length 28.6 ± 0.3 mm and weighing 0.45 ± 0.03 g; *n* = 80, in order to compare the toxicity of silver nanoparticles with silver ions. The nominal exposure concentrations were 13, 21, 23, 25, 29, 37, and 47 *μ*g silver ions L^−1^ administered as silver nitrate plus an unexposed control group.

As an indicator of exposure-related stress, the fish behaviour was assessed by a human observer after 0, 3, 6, 12, 24, 27, 30, and 48 hours of both silver nanoparticles and silver ion treatments. The behavioural components considered were operculum movements (ventilation rate), loss of equilibrium, surface respiration, body colour (pigmentation), circular swimming, jerk movement, bottom resting, and aggressive behaviour. Further, excess mucus production was recorded. Mortality was monitored continuously and fish were considered dead when operculum movement and response to mechanical stimuli could no longer be detected. After termination of the experiment, the remaining fish were killed in ice water and the gender was verified by means of the macroscopic appearance of the gonads.

Following exposure, all water quality values were satisfactory and there were no treatment differences. Oxygen saturation was 91.2 ± 2.5%, pH 6.9 ± 0.1, temperature 25–26°C, and conductivity 280.3 ± 8.4 *μ*S. The load of ammonium, nitrate, and nitrite was measured with Tetra *test* (Tetra Werke, Melle; Germany) and phosphate with JBL test (JBL GmbH & Co. KG, Neuhofen, Germany); Ammonia <0.25 mg L^−1^, nitrate <12.5 mg L^−1^, nitrite <0.3 mg L^−1^, and phosphate 0.25 mg L^−1^.

### 2.5. Statistical Analysis

LC_10_ and LC_50_ values plus their 95% confidence intervals were calculated using the probit analysis in SPSS version 13.0 (SPSS Inc. Chicago, Il, USA).

## 3. Results

### 3.1. Silver Nanoparticle Characterisation

**Figure 1:**
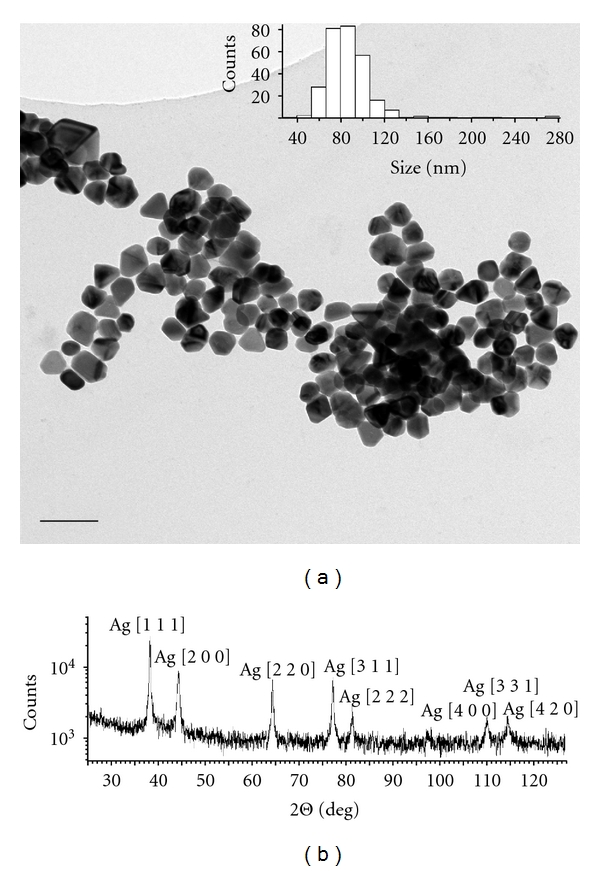
(a) Representative TEM image of stock nanosilver suspension along with size statistics (insert). (b) PXRD pattern for the nanosilver powder with the indexed diffraction lines of silver.

A representative TEM image of the primary silver nanoparticles is shown in Figure 1(a). The nanoparticles were found to predominantly have a slightly elliptical or multifaceted shape, although a few large or triangular particles present. The silver nanoparticle stock preparation had a mean primary size of 81 ± 2 nm and an aspect ratio of 1.2 ± 0.2. The reported size is defined as the maximum distance between two points on the particle circumference, equivalent to the diameter for spherical particles.

The PXRD analysis of the silver nanoparticle powder confirmed the presence of Ag nanocrystallites with a single Ag cubic crystalline orientation (Figure 1(b)) and showed no sign of oxidation or other crystalline phases present. The crystallite domain size estimated by the Sheerer formula was ~78.1 nm.

**Figure 2:**
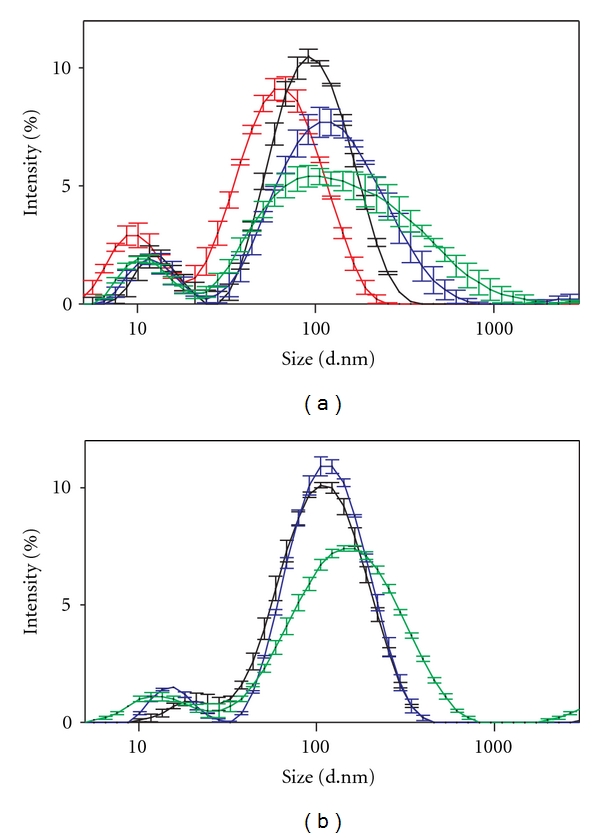
Dynamic Light Scattering (DLS) size distributions for (a) the nanosilver stock preparation (red line) along with the pH 6.9 200 mg L^−1^ NaCl solvent condition 5 min (black line), 1 hour (blue line), and 24 hours (green line). (b) The nanosilver mixed with head tank water directly (1 : 1) (black line), after 30 min (blue line), and 24 hours (green line). Data are presented as mean value ± SE.

As prepared for stock suspension, the silver nanoparticles were characterised using dynamic light scattering (DLS). In general, the DLS analysis presented bimodal distributions, for the stock preparation specifically (Figure 2(a), red curve); the distribution had a major peak at (73.55 ± 1.19) nm (~83% area intensity) and a minor peak around (10.8 ± 0.3) nm (~10% area intensity). In the following, the position of the major peak (73.55 ± 1.19) nm is referenced to as the measured hydrodynamic diameter or size of the nanoparticles under a given set of experimental conditions. Throughout the pH series pH (5–8), the measured silver nanoparticle diameter changed only slightly (~change of −0.1–17 nm), with the offset being evident from the initial mixing (5 min) and then approximately constant throughout the entire time series (5 min, 1 hour, 24 hours) for each pH. The stability of the nanoparticle suspensions under variations in salt concentrations (100–800 mg L^−1^) was tested at two pH values, either as prepared for stock solution (pH 3.9) or equivalent to the head tank water (pH 6.9). For both pH values, the stability of the nanoparticle suspension had pronounced response to salt variations (see Supplementary results, Tables  1(a) and 1(b) available online at doi: 10.1155/2012/293784). At pH 6.9, an initial size increase of ~33–65 nm was observed for all NaCl concentrations, and after 1 h and 24 h, the size continued to increase significantly along with NaCl concentration and time, as exemplified by the pH 6.9 200 mg L^−1^ NaCl experiment in Figure 2(a) after 5 min (black), 1 h (blue) and 24 hours (green). Most pronounced was the increase in hydrodynamic radius of the 600 mg L^−1^ NaCl experiments after 24 hours, reaching (260 ± 29) nm. Besides the increase in size, a temporal development in the width of the DLS size distributions was also observed for all NaCl concentrations (Figure 2(a) and supplementary results, Tables  1(a) and 1(b)). For instance, for the pH 6.9 200 mg L^−1^ experiment (Figure 2(a)), the polidispersity index increased from 0.42 ± 0.02 to 0.507 ± 0.004 ending at 0.54 ± 0.04 for the 5 min, 1 hour, and 24 hours' time points, respectively. At stock preparation pH value 3.9, a likewise trend for the NaCl concentration series was observed concerning the development of the hydrodynamic size with NaCl concentration, reaching a maximum value of (311 ± 9) nm for 800 mg L^−1^ NaCl after 24-hour incubation (supplementary results, Table  1(a)).

Approaching the in vivo conditions, the head tank water preparation (1 : 1) revealed an initial hydrodynamic diameter of (121 ± 3) nm, increasing to (128 ± 3) nm after 30 min ending at (173 ± 3) nm at 24 hours as displayed in Figure 2(b).

In addition to hydrodynamic size measurements, the zeta potential was measured after 24 hours of incubation for the stock nanosilver preparation along with the tested pH and salt variations, including the in vivo relevant head tank water preparation. The stock nanosilver suspension was found to have a zeta potential of (−28.5 ± 0.75) mV, below the head tank water preparations with a zeta potential of (−38.4 ± 0.4) mV. For the variations in pH and salt concentrations, all potentials were measured to be between −27 mV and −48 mV, with no clear correlation between solution conditions and the exact zeta potential value.

### 3.2. Toxicity of Silver Nanoparticles 

**Figure 3:**
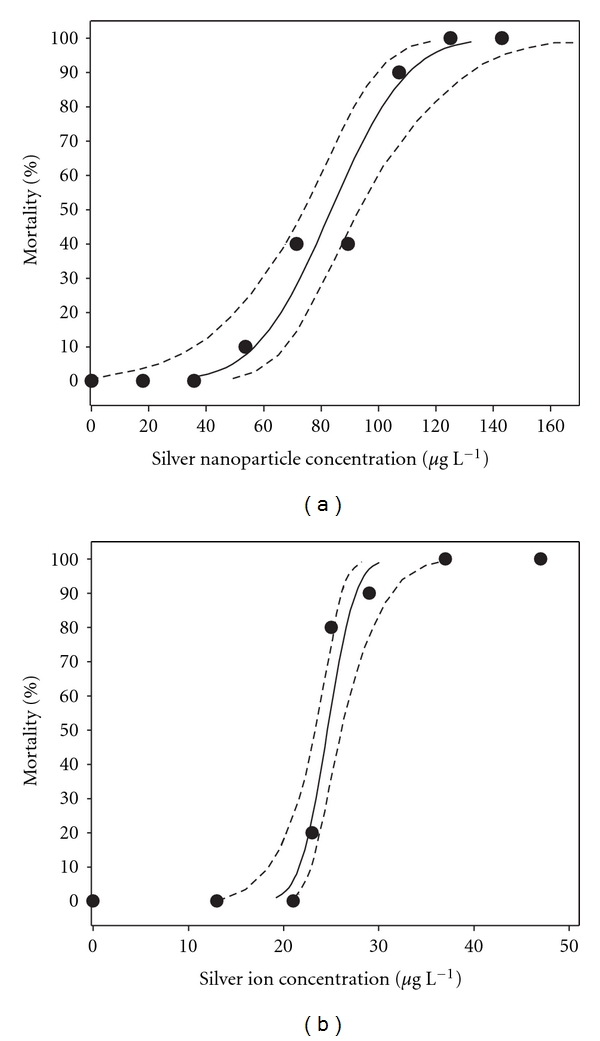
Toxicity of 81 nm silver nanoparticles (a) and silver ions (b) after 48 hours exposure to adult male zebrafish (*Danio rerio)* in a static renewal experiment. Black circles (●) represent the mean survival in percent after exposure to a given concentration (*n* = 10). Solid lines indicate the calculated lethality using probit analysis after exposure to silver nanoparticles and silver ions, respectively. Dashed lines indicate the 95% confidence intervals.

Silver nanoparticles are acutely toxic to male zebrafish (Figure 3(a)) with a semistatic 48-hour exposure LC_50_ of 84 *μ*g L^−1^ (95% CL = 74–93 *μ*g L^−1^) and LC_10_ of 57 *μ*g L^−1^ (95% CL = 36–68 *μ*g L^−1^). A narrow margin was found between the LC_50_ 24 h of 89 *μ*g L^−1^ (95% CL = 79–100 *μ*g L^−1^) and the LC_50_ 48 hour. There was no mortality in the control tank or at the three lowest silver nanoparticle concentrations. After 3 hours of exposure, the first fish started dying, and after 24 hours, all fish in the two highest test concentrations were dead. Extravasations of blood were observed in the anterior ventral surface of the body, just behind the head of the dead fish. This was not found in dead fish exposed to ionic silver.

The toxic action of silver nanoparticles was relatively rapid with signs of stress appearing within 30 min of exposure. At higher silver nanoparticle concentrations (>72 *μ*g L^−1^), toxicity stress signs emerged, starting with zebrafish lying on the tank bottom with increased respiratory rate. Hereafter surface respiration took place, and finally the fish stood still in the middle of the water column where they ultimately lost equilibrium and sank to the bottom. A few fish displayed jerky movements and circular swimming just before they lost equilibrium. No signs of aggressive behaviour or changes in body colour were observed in any of the tanks. After 24 hours of exposure, there were no longer visible differences in behaviour between the control and the lowest exposure groups. Fish mucus (thin white branched threads), likely secreted from the gills, was observed at the bottom of tanks exposed to at least 89 *μ*g L^−1^ silver nanoparticles. Mucus secretion was not observed in the control or the lower concentration exposure tanks.

As expected, no deaths or aberrant behaviour were observed in the groups of fish exposed to PVP alone.

### 3.3. Toxicity of Silver Ions

Mortality increased with increasing concentrations of ionic silver (Figure 3(b)). The 24-hour silver ion LC_50_ was estimated to 28 *μ*g L^−1^ (95% CL = 26–31 *μ*g L^−1^) and the estimated 48-hour LC_50_ was 25 *μ*g L^−1^ (95% CL = 23–26 *μ*g L^−1^). No mortality was recorded in the two lowest concentrations or in the control group. Signs of stress were revealed by the fish displaying increased swimming activity and attempts to escape from the tank. Behavioural stress signs were the same as described above for silver nanoparticles. In addition, elevated mucus secretion with strands of sloughed mucus appeared in the tank after exposure to higher concentrations of silver ions.

## 4. Discussion

### 4.1. Silver Nanoparticle Characterisation

The nanoparticle characterisation was conducted using a range of complementary techniques in order to characterise both the primary nanoparticle size distribution; and the in-suspension agglomeration under varying aqueous conditions.

The primary size was determined by image analysis on a representative selection of TEM images. Statistically, the TEM analysis demonstrated both a larger size and a particle morphology differing from the nominal spherical 30–40 nm particles. In line with previous work on a larger range of metal nanoparticles [27] this emphasises the importance of an in-house size characterisation for nanotoxicology studies.

The primary nanoparticle is not necessarily homogeneous in terms of chemical or crystalline properties. Metallic nanoparticles samples can contain both amorphous and various crystalline phases in elaborate geometrical configurations (e.g., core-shell), and nanoparticle materials can additionally be affected by postsynthesis oxidation. The PXRD analysis for the silver nanoparticles was consistent with a single Ag cubic crystalline phase present and showed no sign of oxidation or other crystalline phases. The estimate of crystallite size provided by the FWHM Scheerer formula is only accurate in the absence of additional contributions to the observed line broadening, like lattice strain caused by; for example, point defects or plastic deformations [28]. Additionally, it is important to emphasise that crystallite size is not generally the same as particle size, but only a measure of the size of a coherently diffracting domain; this is why we rely on TEM and DLS accurate particle sizing in the presented work. However, while a full analysis addressing microstrain is beyond the scope of this text, the agreement between the TEM primary partice size characterization and the Scheerer estimate of crystallite size suggests that the nanosilver particles have a good degree of crystallinity, with a limited amorphous component only.

Determining particle sizes via TEM or PXRD facilitates insight to the primary and crystalline sizes, but the techniques do not bring knowledge on how the nanoparticles agglomerate or behave in suspension under relevant aqueous conditions, which may affect aggregation significantly. A key element to investigating the biological response to nanoparticle exposure via liquid delivery is the ability to actually have free nanoparticles in suspension, as quantified by the difference between the primary nanoparticle size and in-suspension agglomeration and resulting size-distribution. The nanosilver utilised in the present study was synthesised with 0.2% PVP. PVP is a general metal-oxide chelating agent, allowing a strong binding to the silver nanoparticles while stabilising the particle suspension and redispersion by steric repulsion [29]. The stock preparation of silver nanoparticles had a bimodal size distribution; yet no clear evidence of a bimodal distribution was found by TEM. We thus attribute the minor peak at ~11 nm to scattering on free PVP in suspension in correspondence with DLS studies on PVP polymers [30]. The peak hydrodynamic size of the stock silver suspension was in good correspondence with the established TEM size statistics, signifying an only limited agglomeration of the silver nanoparticles when prepared following our specific redispersion protocol. This limited aggregation is in good agreement with the absolute value of the zeta potential measurements, as absolute zeta potentials in the range or above 30 mV indicate particle agglomeration stability.

During an in vivo experiment, both the ionic strength and the pH of the water will differ from those of the stock silver nanoparticle suspension and potentially change over time. The pH value of head tank water was measured to be pH 6.9; this is higher than the pH 3.9 observed for the stock preparation; this is why the nanoparticle suspension was subjected to a pH (5–8) variation test. The eight most abundant ionic species in the utilised head tank water preparation (SO_4_^2−^, Na^+^, NO_3_^−^, Mg^2+^, K^+^, Cl^−^, Ca^2+^, HCO_3_^−^) have a total concentration of ~137 mg L^−1^. The effective screening of these eight predominant ions can be quantified by the Debye length [31] with each ionic species, however, having a unique impact on the effective Debye length depending on both their specific molar concentration and charge. The combined Debye length was calculated as 6.3 nm, with clear dominating contributions from Na^+^ and Cl^−^. The silver nanoparticles were, therefore, subjected to a salt solution series of (100–800) mg L^−1^ NaCl, with 138 mg L^−1^ NaCl constituting a head tank water equivalent at a Debye length of 6.3 nm, situated reasonably between the 100 mg L^−1^ and 200 mg L^−1^ measurements. Throughout the pH series, only a minor change in the nanoparticle hydrodynamic size was observed, signifying that the PVP encapsulation and steric repulsion is stable against pH variations. For the variations in salt (100–800) mg L^−1^, however, both an initial and temporal increase in size occurred. Synthesised silver nanoparticle suspensions without the addition of stabilisers, like PVP, are susceptible to agglomeration upon an increase in salt concentrations. The observed agglomeration, with a maximum at 600 mg L^−1^ pH 6.9, indicates defects and/or incomplete coverage of the PVP encapsulation. The fact that the peak-agglomerated size is only 3-4-fold the peak size of the stock suspension demonstrates, however, that the agglomeration is limited even in this worst case scenario.

For the in vivo relevant head tank water conditions, the peak-agglomerated size represented a 2-3-fold increase compared with stock conditions, thus performing better than the above described worst case scenario. The measured DLS size distribution agreed well with the corresponding pH value and ionic screening conditions (pH 6.9; 200 mg L^−1^), both strengthening the DLS head tank water results and suggesting that the pH and salt solution series provide a valid insight to the behaviour of the silver nanoparticles under in vivo conditions.

Measured after 24 hours, the zeta potentials of the varying aqueous conditions (pH, salt variations, and head tank water) were all below −27 mV agreeing well with the observed limited agglomeration, but also indicating that some steady state or equilibrium size distribution was reached within the 24-hour observation time.

Concerning variations in pH, ionic concentrations, and dispersion in tank water, the nanoparticle characterisation shows the silver nanoparticles to have good stability against agglomeration, ensuring a population of free nanosized particles at the relevant time-scale of the in vivo experiments. For the initial exposure of silver nanoparticles, we thus argue that the current characterisation gives an adequate description of the silver nanoparticle particle distribution during the LC_50_ assessments.

### 4.2. Acute Toxicity of Silver Nanoparticles and Silver Ions

The present study demonstrates that the silver nanoparticles used are acutely lethal to adult zebrafish. Previously, the 48-hour nanosilver LC_50_ value has been found to be 7.07 mg L^−1^ in zebrafish exposed to metal oxide-coated silver nanoparticles with a size of 44.5 and 216 nm in suspension [14]. In another study on zebrafish, Choi et al. [32] reported the 24-hour LC_50_ to be 250 mg L^−1^ for 5–20 nm silver nanoparticles, which is far less toxic than the LC_50_ value found by Griffitt et al. [14] and in the present study. In Japanese medaka (*Oryzias latipes*), the 96-hour LC_50_ has been demonstrated to be 34.6 *μ*g L^−1^ for 50 nm uncoated silver particles [33]. Accordingly, it is clear that silver nanoparticles of different size and with or without different stabilisation agents process different degrees of toxicity in different organisms, under different exposure times and conditions.

Dynamic light scattering showed that the intensity of silver nanoparticles in the tank water decreased after 24 hours, indicating a concentration decline. Also, AAS measurements of water samples revealed a decline in silver concentration after 24 hours. This decline is likely due to particle adhesion to the sides of the tank. Accordingly, the actual silver nanoparticle exposure concentration available to the fish is presumably lower than the reported nominal concentration. This can explain that the reverted behaviour where the fish exposed to the lowest nanosilver concentrations after 24 hours resembled the behaviour of the control fish.

The demonstrated toxicity of the silver nanoparticles was attributed to the silver and not the PVP coating. Besides, PVP has successfully been applied as a nontoxic additive in a broad range of products, such as shampoo, toothpaste, contact lens solution, as a binder in pharmaceutical tablets, and as a food additive stabilizer.

As expected, the results of the present study confirmed that ionic silver is also acutely toxic to zebrafish at concentrations consistent with a prior estimation for zebrafish [14] and within the 96-hour LC_50_ range (5–70 *μ*g L^−1^) for other teleosts [8]. Given that tank water contained NaCl, it cannot be excluded that some of the silver existed as silver chloride. In rainbow trout (*Oncorhyncus mykiss*), Hogstrand et al. [34] found that dissolved silver chloride is at least ten times less toxic than silver ions to fish and particulate silver chloride is nontoxic. Similarly, Bury et al. [35] reported that increasing chloride concentrations decreased silver toxicity in rainbow trout whereas chloride ions did not significantly affect the 96-hour LC_50_ values for fathead minnows (*Pimephales promelas*).

The silver ions were approximately 3.4 times more toxic than the silver nanoparticles by mass of silver added to the tanks. In an in vitro study, PVP-coated nanosilver from the same supplier as the particles in the present study was found to be approximately four times less toxic than silver ions [36], indicating that nanoparticulate forms of silver are less toxic than their soluble forms by mass added. As with silver, this has also been shown to be the case for copper [14, 15] and aluminium nanoparticles [14]. In contrast, in a study on juvenile Japanese medaka (*Oryzias latipes*) Chae et al. [33] reported that nanosilver was more toxic than ionic silver after 24 hours of exposure and similar in toxicity after 96 hours.

The fraction of nanosilver that is chemically available is worth considering. If the nanosilver toxicity is caused by chemical interactions, the toxic portion of nanoparticles must originate either from silver ions dissolved from the particle or from the exposed silver atoms on the particle surface. For 80 nm particles using the bulk silver density and a hexagonal packing of 0.29 diameter silver atoms, the percentage of surface atoms is ~0.2%. Thus, the vast majority of the silver (>99.8%) is expected to be contained within the core of the nanoparticles. Silver ions can be released from the surface of silver nanoparticles [37] and the ISE and ultracentrifugation methods surprisingly estimated that up to 40% of the silver by mass existed as ionic silver in the nanosilver suspension. In contrast, Navarro et al. [22] estimated that 1% of the silver in a carbonate-coated nanosilver suspension was free silver ions. This underlines the importance of estimating the dissolution of metal nanoparticles. In fathead minnow embryo, the 96-hour LC_50_ of nanosilver was lower than the 15 *μ*g L^−1^ silver nitrate LC_50_ value, even though the amount of dissolved silver from nanosilver was 18–95 *μ*g L^−1^ [38]. Laban et al. [38] therefore presented the idea that silver ions dissociated from silver nitrate process a different toxicity than silver ions released from silver nanoparticles. In fact, this is in accordance with our results, because the silver ion LC_50_ value is below the amount of dissolved silver from the nanoparticles. Furthermore, only zebrafish exposed to silver ions as silver nitrate displayed an avoidance reaction, which was not observed in nanosilver exposed fish. Silver ions most likely contributed to the toxicity of the nanoparticle suspension. On the other hand, nanoparticles undoubtedly have an inherent toxicity. For example, extravasations of blood were only observed in fish exposed to silver nanoparticles. The precise mechanism whereby silver nanoparticles exert their toxicity to fish is to our knowledge unknown. The observed increased ventilation rate, surface respiration, and excessive gill mucus secretion during nanosilver exposure suggest the gills as target organs of nanosilver toxicity. Likewise, during ionic silver exposure, hyperventilation was observed in rainbow trout (*Oncorhynchus mykiss*) and attributed to counteracting metabolic acidosis [39]. Increased mucus secretion after ionic silver application has previously been noticed [40]. When a toxicant irritates the gill epithelium, mucus production is increased, trapping and transporting the toxicant away from the gills [18]. However, excessive mucus secretion also increases the oxygen diffusion distance, impairing respiration. Other nanoparticles, such as carbon nanotubes, have caused increased mucus secretion [41] and swollen mucocytes have been induced by titanium dioxide [42]. The primary target of silver ion acute toxicity for freshwater fish is, on the other hand, known to be the gills, where ionic silver accumulates and binds to the gill epithelium. These bindings lead to inhibition of Na^+^ and K^+^-ATPase activity, whereby the active uptake of Na^+^ and Cl^−^ is inhibited [13, 43, 44]. Such ion regulatory disturbances may ultimately be lethal to the fish [43]. Additionally, it has been suggested that silver ions partially inhibit the carbonic anhydrase enzyme, which catalyze the hydration of CO_2_ to produce H^+^ and HCO_3_^−^, which may be exchanged for external Na^+^ and Cl^−^ [45]. This is, however, not the primary mechanism of silver toxicity in fish [46]. Although it appears that both nanosilver and ionic silver exert their toxicity in the gills of fish, different mechanisms of toxicity are probably in play. For instance, extravasations of blood were only observed in fish exposed to nanosilver. In gram-negative bacteria, the overall effect of nanosilver also differs from the effect of silver ions [37].

The zebrafish sensed the silver ions in the water displaying an avoidance reaction, where they increased their swimming activity and were trying to escape out of the tank. This behaviour was not observed among the controls or the nanosilver-exposed fish. Most likely, the 81 nm silver nanoparticles are too big to be sensed by olfaction as opposed to the silver ions with an ionic radius of 0.137 nm [1]. A behavioural avoidance response was also observed in rainbow trout exposed to copper, cobalt [47], and chromium [48]. It has been suggested that olfactory receptors detect copper in the water [47], which may also be true of ionic silver.

The LC_50_ value of silver nanoparticles estimated in the present study is higher than present concentrations in the aquatic environment [9]. However, point discharges may result in significantly higher local nanosilver concentrations, and the expected increase in the usage of nanosilver in the nearest future will undoubtedly result in increased concentrations in the aquatic environment.

## Supplementary Material

Table 1: Silver nanoparticle size by Dynamic Light Scattering (DLS). The used silver nanoparticles were coated with 0.2% polyvinyl pyrrolidone (PVP) (stated by the manufacturer to be spherical 30–40 nm particles). Particle suspensions were subjected to sodium chloride contractions (100 mg L^−1^ to 800 mg L^−1^) at stock pH value 3.9 and at the head tank (fish tank water) equivalent pH value 6.9, followed over 24 hours at 26°C. The samples were kept between measurements under conditions identical to the fish tanks used for nanosilver toxicity assessment. DLS measurement was run in triplicate using automated, optimal measurement time and laser attenuation settings.
