# Supplementary material for: In Vivo Toxicity of Silver Nanoparticles and Silver Ions in Zebrafish (Danio rerio)
Source: J Toxicol. 2011 Dec 1;2012:293784. doi: 10.1155/2012/293784 (PMC3235887; doi:10.1155/2012/293784)
Supplement: Supplementary file 1 — Table 1: Silver nanoparticle size by Dynamic Light Scattering (DLS). The used silver nanoparticles were coated with 0.2% polyvinyl pyrrolidone (PVP) (stated by the manufacturer to be spherical 30–40 nm particles). Particle suspensions were subjected to sodium chloride contractions (100 mg L−1 to 800 mg L−1) at stock pH value 3.9 and at the head tank (fish tank water) equivalent pH value 6.9, followed over 24 hours at 26°C. The samples were kept between measurements under conditions identical to the fish tanks used for nanosilver toxicity assessment. DLS measurement was run in triplicate using automated, optimal measurement time and laser attenuation settings. [file 293784.f1.pdf]

Table 1A, pH value 3.9

**Sample**	**Size[a] (SE) [nm]**	**PDI[b] (SE)**
100 mg 5min	101 (2)	0.286 (0.002)
200 mg 5min	103 (1)	0.286 (0.009)
400 mg 5min	114 (2)	0.294 (0.002)
600 mg 5min	108 (1)	0.39 (0.01)
800 mg 5min	108 (1)	0.32 (0.05)
100 mg 1-2h	136 (5)	0.449 (0.008)
200 mg 1-2h	137 (2)	0.41 (0.02)
400 mg 1-2h	151 (1)	0.303 (0.002)
600 mg 1-2h	182 (1)	0.306 (0.005)
800 mg 1-2h	204 (4)	0.286 (0.008)
100 mg 24h	143 (1)	0.482 (0.006)
200 mg 24h	158 (9)	0.45 (0.04)
400 mg 24h	210 (4)	0.39 (0.06)
600 mg 24h	269 (3)	0.386 (0.008)
800 mg 24h	311 (9)	0.30 (0.01)

a) Peak position of the major peak in the DLS distribution (Standard Error)

b) Polydispersity index calculated by the Malvern Dispersion Software (Standard Error)

Table 1B, pH value 6.9

**Sample**	**Size[a] (SE) [nm]**	**PDI[b] (SE)**
100 mg 5min	111 (6)	0.38 (0.06)
200 mg 5min	106 (3)	0.42 (0.02)
400 mg 5min	111 (3)	0.42 (0.01)
600 mg 5min	116 (9)	0.43 (0.03)
800 mg 5min	138 (14)	0.46 (0.03)
100 mg 1-2h	137 (4)	0.498 (0.004)
200 mg 1-2h	148 (8)	0.507 (0.004)
400 mg 1-2h	170 (3)	0.55(0.008)
600 mg 1-2h	165 (1)	0.53(0.01)
800 mg 1-2h	193 (4)	0.58 (0.01)
100 mg 24h	170 (15)	0.6 (0.2)
200 mg 24h	205 (21)	0.54 (0.04)
400 mg 24h	202 (16	0.58 (0.05)
600 mg 24h	260 (29)	0.554 (0.008)
800 mg 24h	196 (7)	0.62 (0.09)

[a)] Peak position of the major peak in the DLS distribution

[b)] Polydispersity index calculated by the Malvern Dispersion Software
